# In-Hospital Mortality Disparities Among American Indian and Alaska Native, Black, and White Patients With COVID-19

**DOI:** 10.1001/jamanetworkopen.2022.4822

**Published:** 2022-03-30

**Authors:** Leslie A. Musshafen, Lamees El-Sadek, Seth T. Lirette, Richard L. Summers, Caroline Compretta, Thomas E. Dobbs

**Affiliations:** 1 Department of Population Health Science, John D. Bower School of Population Health, University of Mississippi Medical Center, Jackson; 2 Mississippi State Department of Health, Jackson; 3 Department of Data Science, John D. Bower School of Population Health, University of Mississippi Medical Center, Jackson; 4 Department of Emergency Medicine, University of Mississippi Medical Center, Jackson; 5 Department of Preventive Medicine, John D. Bower School of Population Health, University of Mississippi Medical Center, Jackson

## Abstract

**Importance:**

American Indian and Alaska Native populations have some of the highest COVID-19 hospitalization and mortality rates in the US, with those in Mississippi being disparately affected. Higher COVID-19 mortality rates among Indigenous populations are often attributed to a higher comorbidity burden, although examinations of these associations are scarce, and none were believed to have included individuals hospitalized in Mississippi.

**Objective:**

To evaluate whether racial mortality differences among adults hospitalized with COVID-19 are associated with differential comorbidity experiences.

**Design, Setting, and Participants:**

The described cross-sectional study used retrospective hospital discharge data from the Mississippi Inpatient Outpatient Data System. All adult (aged ≥18 years) Mississippians of a known racial identity and who had been hospitalized with COVID-19 from March 1 to December 31, 2020, in any of the state’s 103 nonfederal hospitals were included. Data were abstracted on June 17, 2021.

**Exposure:**

Racial identity.

**Main Outcomes and Measures:**

In-hospital mortality as indicated by discharge status.

**Results:**

A total of 18 731 adults hospitalized with a COVID-19 diagnosis and known racial identity were included (median age, 66 [IQR, 53-76] years; 10 109 [54.0%] female; 225 [1.2%] American Indian and Alaska Native; 9191 [49.1%] Black; and 9121 [48.7%] White). Pooling across comorbidity risk groups, odds of in-hospital mortality among Black patients were 75% lower than among American Indian and Alaska Native patients (odds ratio [OR], 0.25 [95% CI, 0.18-0.34]); odds of in-hospital death among White patients were 77% lower (OR, 0.23 [95% CI, 0.16-0.31]). Within comorbidity risk group analyses, Indigenous patients with the lowest risk (Elixhauser Comorbidity Index score ≤0) had an adjusted probability of in-hospital death of 0.10 compared with 0.03 for Black patients (OR, 0.29 [95% CI, 0.10-0.82]) and 0.04 for White patients (OR, 0.37 [95% CI, 0.13-1.07]). Probability of in-hospital death at the highest comorbidity risk levels (Elixhauser Comorbidity Index score ≥16) was 0.69 for American Indian and Alaska Native patients compared with 0.28 for Black patients (OR, 0.16 [95% CI, 0.08-0.32]) and 0.25 for White patients (OR, 0.14 [95% CI, 0.07-0.27]).

**Conclusions and Relevance:**

This cross-sectional study of US adults hospitalized with COVID-19 found that American Indian and Alaska Native patients had lower comorbidity risk scores than those observed among Black or White patients. Despite empirical associations between reduced comorbidity risk scores and reduced odds of inpatient mortality, American Indian and Alaska Native patients were significantly more likely to die in the hospital of COVID-19 than Black or White patients at every level of comorbidity risk. Alternative factors that may contribute to high mortality rates among Indigenous populations must be investigated.

## Key Points

**Question:**

Are the higher in-hospital mortality rates for COVID-19 among American Indian and Alaska Native patients compared with other racial groups associated with differences in comorbidity burden?

**Findings:**

In this cross-sectional study of 18 731 US adults hospitalized with COVID-19 in 2020, American Indian and Alaska Native patients had a lower mean comorbidity risk score compared with the overall patient population, yet they were significantly more likely than patients of all other races to die in the hospital. Increased COVID-19 in-hospital deaths among American Indian and Alaska Native adults were not associated with increased comorbidity experiences in all populations.

**Meaning:**

These findings suggest that alternative factors contributing to disparate in-hospital mortality rates among Indigenous communities must be investigated further.

## Teaser

This cross-sectional study assesses comorbidity risks and in-hospital outcomes of American Indian and Alaska Native patients with COVID-19 compared with patients of other races and ethnicities.

## Introduction

The COVID-19 pandemic has disparately affected racial and ethnic minority groups in the US, putting these groups at great risk of infection, hospitalization, and death due to the novel coronavirus.^1,2,3^ Indigenous populations are believed to be one of the worst affected in the nation. As of November 22, 2021, American Indian and Alaska Native persons were 1.6 times more likely to have SARS-CoV-2 infection, 3.3 times more likely to be hospitalized, and 2.2 times more likely to die as a result of COVID-19 than non-Hispanic White persons.^4^ As of December 15, 2021, a reported 296 967 infections^5^ and 8983 deaths^6^ of American Indian and Alaska Native individuals were attributed to the novel coronavirus in the US.

In Mississippi, COVID-19 spread rapidly throughout the only federally recognized American Indian tribe in the state, the Mississippi Band of Choctaw Indians. One in 10 of the nearly 10 000 members of this tribe contracted the virus within the first 6 months of its appearance in the state, compared with 1 in 50 of all residents who had become infected statewide within this same period.^7,8^ Although American Indian and Alaska Native individuals accounted for less than 1% of the state’s population, they represented 4.5% of COVID-19 deaths in the state by July 2020.^9^

Long-standing issues related to racial misclassifications and missing Indigenous population data within public health and empirical records have been highlighted by the COVID-19 pandemic. The first report by the Centers for Disease Control and Prevention including American Indian and Alaska Native population experiences with the virus, for instance, came 5 months into the pandemic and included information from only 23 states.^10^ A recent systematic review of racial and ethnic disparities in COVID-19–related infections, hospitalizations, and fatalities reported a similar dearth of data, with only 9 of 52 related studies including American Indian and Alaska Native individuals; in no instance did Indigenous individuals exceed 1% of a total study population.^3^

Higher mortality rates among American Indian and Alaska Native individuals as a result of COVID-19 have been widely attributed to a higher prevalence of comorbidities among Indigenous populations.^11,12^ A 2020 Kaiser Family Foundation study found a greater proportion of American Indian and Alaska Native adults at high risk of severe illness as a result of COVID-19 infection than adults of all other races and ethnicities given increased rates of asthma, chronic obstructive pulmonary disease, diabetes, heart disease, and obesity.^12^ In Mississippi, a state often ranked at the bottom of US health ratings, these disparities persist. Compared with the state’s general population, American Indian and Alaska Native Mississippians experience higher rates of chronic conditions for all measures with Indigenous populations data, including asthma, cardiovascular disease, diabetes, and smoking.^13^

The Elixhauser Comorbidity Index (ECI) is one of the most widely used measures of comorbidity risk today. Originally introduced in 1998, the ECI calculates a person’s risk for various health outcomes based on the presence of comorbid conditions.^14^ An algorithm was developed by van Walraven et al^15^ in 2009 to assign validated risk weights to each of 30 or 31 ECI chronic conditions, producing a single risk score for each individual such that higher ECI scores are associated with increased odds of death. This weighted approach has proven superior to unweighted ECI scores or other indexes at reducing type I errors and predicting odds of in-hospital mortality.^16,17^ Application to COVID-19 interrogations have also proven effective.^18,19,20^

A recent study in a single Mississippi hospital^21^ found that even when controlling for the risk factors that the Centers for Disease Control and Prevention believed at the time put individuals at greatest risk of death from the novel coronavirus, American Indian and Alaska Native adults with COVID-19 had an in-hospital odds of death more than twice that of Black adults (odds ratio [OR], 0.23; *P* < .001) or White adults (OR, 0.24; *P* < .001). The authors therefore hypothesized that COVID-19 mortality rates would be significantly greater among American Indian and Alaska Native adults than those of any other race admitted to hospitals across the state and that higher COVID-19 in-hospital mortality rates among Indigenous patients would not be associated with greater comorbidity risk among American Indian and Alaska Native patients. To our knowledge, our study is the first to evaluate the associations between ECI comorbidity risk and adult COVID-19 mortality outcomes across races within Mississippi hospitals. This study aimed to fill substantial gaps in the literature with regard to COVID-19 comorbidity risks and in-hospital outcomes among American Indian and Alaska Native patients.

## Methods

### Study Design and Settings

This cross-sectional study was conducted in coordination with the Mississippi State Department of Health using retrospective data from the Mississippi Inpatient Outpatient Data System, the state’s hospital discharge data repository.^22^ The Mississippi Inpatient Outpatient Data System contains deidentified data extracted from the electronic health records of all nonfederal, acute, and long-term hospitals in the state. The Human Research Office at the University of Mississippi Medical Center determined that institutional review board approval was not needed because the requested data set did not contain patient identifiers and the investigators did not seek to identify patients or extrapolate identifiable patient data. A data use agreement was executed by all investigators who would have access to the data to define terms of use. We followed the Strengthening the Reporting of Observational Studies in Epidemiology (STROBE) guidelines for reporting cross-sectional studies.

### Data Abstraction and Variables of Interest

All adults (aged ≥18 years) hospitalized with COVID-19 at any nonfederal hospital in Mississippi in 2020 with a known racial identity and a self-reported address in the state were included. All patients with an unknown racial identity (eg, patient declined or unavailable) or self-reported residence outside of Mississippi were excluded. In accordance with the Centers for Disease Control and Prevention’s official coding guidelines,^23^ COVID-19 hospitalizations were identified using *International Statistical Classification of Diseases and Related Health Problems, Tenth Revision* (*ICD-10*), code B97.29 (other coronavirus as the cause of diseases classified elsewhere) from March 1 through April 30, 2020, or code U07.1 (COVID-19 virus identified) from April 1 through December 31, 2020, when the distinct code for COVID-19 was established and adopted. For all patients meeting inclusion criteria, age, sex, race and ethnicity, admission/discharge dates, length of stay, discharge status, and all defined *ICD-10* codes associated with any of the 31 ECI comorbid conditions (eTable in the Supplement) were abstracted from the Mississippi Inpatient Outpatient Data System data set on June 17, 2021.

The in-hospital mortality outcome of interest was derived by collapsing 37 possible patient discharge statuses into a binary alive or deceased indicator. Only 1 status (expired) was used to capture those who died while in the hospital; all others (eg, transferred to another facility, discharged, left against medical advice) are reported as alive at discharge. Risk factors of interest included race (American Indian and Alaska Native, Asian, Black, Native Hawaiian or other Pacific Islander, White, or multiracial [≥2 races]), age (continuous variable), sex (male or female), and weighted ECI score. Patient age, sex, and race were self-reported, reported by individuals accompanying the patient, or ascertained by medical personnel. All variable categories are reported as they appear in the Mississippi Inpatient Outpatient Data System.

Unique identification numbers were used to screen all 2020 admission records of included patients for *ICD-10* codes associated with any of the 31 ECI comorbid conditions. A binary indicator (0 or 1) was generated to account for the absence or presence of each condition within included individuals. The scoring algorithm of van Walraven et al,^15^ based on each condition’s weighted association with death, was then applied to produce a single ECI risk score for each patient. Scores were then grouped to stratify mortality risks into 5 categories: 0 or less, 1 to 5, 6 to 10, 11 to 15, and 16 or greater.

### Statistical Analysis

Demographic and clinical characteristics of the patient cohort were derived using descriptive statistics. For each race represented, characteristics were further stratified by sex, age, ECI risk score group, and patient discharge status. Univariate and multivariate logistic regression models were run to assess associations of race, comorbid conditions (ECI group), age, and sex with the in-hospital mortality outcome of interest. Wald χ^2^ tests were used to assess each variable. Univariate regression models were evaluated using χ^2^ tests. The full multivariate model included age, sex, race, and ECI group as variables of interest with the in-hospital mortality outcome, given empirical evidence that each may impact inpatient death. Variance inflation factors were used to test for multicollinearity among independent regression variables. All statistical analyses were conducted using Stata/SE, version 17.0 (StataCorp LLC); *P* < .05 indicated statistical significance.

## Results

### Study Population Demographic and Clinical Characteristics

A total of 18 731 adult Mississippians with a COVID-19 diagnosis and known race were admitted across 103 nonfederal hospitals in the state in 2020 (Table 1); 35 otherwise eligible patients without a known race were excluded. Among the included patients, 10 109 (54.0%) were female and 8622 (46.0%) were male. Cohort patient ages ranged from 18 to 110 years, with a median age of 66 (IQR, 53-76) years. Among adults hospitalized with COVID-19, American Indian and Alaska Native patients were significantly younger (mean [SD] age, 52.4 [16.8] years) than Black (mean [SD] age, 60.2 [16.8] years) or White (mean [SD] age, 68.3 [15.6] years) patients. Most patients self-identified as Black (9191 [49.1%]) or White (9121 [48.7%]). Additional racial identities included American Indian and Alaska Native (225 [1.2%]), Asian (64 [0.3%]), Native Hawaiian or other Pacific Islander (18 [0.1%]), and 2 or more races (112 [0.6%]). Among those reporting an ethnic origin, 18 196 (97.1%) self-identified as non-Hispanic or non-Latino. American Indian and Alaska Native patients had the highest mean (SD) length of stay time at 9.9 (9.8) days compared with 8.0 (9.5) days among Black patients and 7.8 (9.6) days among White patients.

**Table 1.** Demographic and Clinical Characteristics of Adult Patients With COVID-19 and a Known Race in All Nonfederal Mississippi Hospitals in 2020

Characteristic	Race^a^						
	American Indian and Alaska Native (n = 225)	Asian (n = 64)	Black (n = 9191)	Native Hawaiian or other Pacific Islander (n = 18)	White (n = 9121)	≥2 Races (n = 112)	All (N = 18 731)
Sex							
Female	127 (56.4)	27 (42.2)	5314 (57.8)	11 (61.1)	4579 (50.2)	51 (45.5)	10 109 (54.0)
Male	98 (43.5)	37 (57.8)	3877 (42.2)	7 (38.9)	4542 (49.8)	61 (54.5)	8622 (46.0)
Age, y							
Mean (SD)	52.4 (16.8)	61.3 (15.3)	60.2 (16.8)	50.3 (14.3)	68.3 (15.6)	51.2 (17.1)	64 (16.8)
Median (IQR)	53 (40-64)	63 (53-72)	62 (49-72)	51 (38-60)	71 (59-80)	47 (39-63)	66 (53-76)
Weighted ECI score^b^							
Mean (SD)	7.8 (8.3)	7.1 (7.7)	8.1 (9.2)	6.8 (7.3)	8.2 (8.7)	4.8 (7.2)	8.1 (8.9)
≤0	58 (25.8)	15 (23.4)	2344 (25.5)	4 (22.2)	2094 (23.0)	40 (35.7)	4555 (24.3)
1-5	54 (24.0)	26 (40.6)	2144 (23.3)	8 (44.4)	2313 (25.3)	36 (32.1)	4581 (24.5)
6-10	36 (16.0)	6 (9.4)	1523 (16.7)	2 (11.1)	1550 (17.0)	17 (15.2)	3134 (16.7)
11-15	38 (16.9)	8 (12.5)	1241 (13.5)	1 (5.5)	1341 (14.7)	9 (8.0)	2638 (14.1)
≥16	39 (17.3)	9 (14.1)	1939 (21.1)	3 (16.7)	1823 (20.0)	10 (8.9)	3823 (20.4)
Length of stay, d							
Mean (SD)	9.9 (9.8)	5.9 (6.0)	8.0 (9.5)	6.7 (7.2)	7.8 (9.6)	8.7 (10.4)	7.9 (9.5)
Median (IQR)	6 (3-13)	4 (2-7.5)	5 (3-9)	4.5 (2-7)	5 (3-9)	5 (3-9.5)	5 (3-9)
Patient status							
Alive	158 (70.2)	55 (85.9)	7941 (86.4)	16 (88.9)	7789 (85.4)	104 (92.9)	16 063 (85.7)
Deceased	67 (29.8)	9 (14.1)	1250 (13.6)	2 (11.1)	1332 (14.6)	8 (7.1)	2668 (14.2)

Abbreviation: ECI, Elixhauser Comorbidity Index.

^a^ Unless otherwise indicated, data are expressed as number (%) of patients. Percentages are rounded and may not total 100.

^b^ Higher scores indicate more comorbid disease burden.

### Comorbidity Experiences

Weighted ECI scores for the overall cohort ranged from −14 to 58; among American Indian and Alaska Native patients, ECI scores ranged from −7 to 39. American Indian and Alaska Native patients had a lower mean (SD) comorbidity risk score (7.8 [8.3]) than that of the overall cohort (8.1 [8.9]), Black patients (8.1 [9.2]), or White patients (8.2 [8.7]) (Table 1). Among all included patients, 9136 (48.8%) had a weighted ECI score of 5 or less and 9595 (51.2%) had a score of 6 or greater. Among all inpatients with a COVID-19 diagnosis, 3823 (20.4%) were in the highest comorbidity risk bracket with an ECI score of 16 or greater. Proportionally more American Indian and Alaska Native patients had the lowest ECI scores of less than 0 to 5 (112 [50.0%]) and fewer had the highest ECI score of 16 or greater (39 [17.3%]) compared with Black (4488 [48.8%] and 1939 [21.1%], respectively) and White (4407 [48.3%] and 1823 [20.0%], respectively) patients.

Across the 31 ECI comorbid conditions, proportionally fewer American Indian and Alaska Native patients experienced 22 conditions compared with Black or White patients (Table 2). American Indian and Alaska Native patients had the greatest rates of fluid and electrolyte disorders (127 [56.4%]), diabetes (58 [25.8%] for uncomplicated and 99 [44.0%] for complicated), kidney failure (61 [27.1%]), coagulopathy (47 [20.9%]), liver disease (20 [8.9%]), rheumatoid arthritis or other collagen vascular diseases (10 [4.4%]), and alcohol (9 [4.0%]) and drug (4 [1.8%]) abuse. In addition, more than one-quarter of American Indian and Alaska Native patients were diagnosed with hypertension (uncomplicated in 87 [38.7%] and complicated in 65 [28.9%]) and obesity (61 [27.1%]).

**Table 2.** Elixhauser Comorbidity Index Risk Factors Among Adults With COVID-19 and a Known Race Admitted to Any Nonfederal Mississippi Hospital, 2020

Risk factor	Race, No. (%)			
	American Indian and Alaska Native (n = 225)	Black (n = 9191)	White (n = 9121)	All (N = 18 731)^a^
AIDS/HIV	0	37 (0.4)	2 (0.01)	39 (0.2)
Alcohol abuse	9 (4.0)	164 (1.8)	113 (1.2)	289 (1.5)
Blood loss anemia	1 (0.4)	48 (0.5)	27 (0.3)	77 (0.4)
Cardiac arrhythmias	41 (18.2)	1755 (19.1)	2560 (28.1)	4391 (23.4)
Chronic pulmonary disease	13 (5.8)	1620 (17.6)	2165 (23.7)	3812 (20.3)
Coagulopathy	47 (20.9)	1155 (12.6)	924 (10.1)	2143 (11.4)
Congestive heart failure	27 (12.0)	1930 (21.0)	1722 (18.9)	3697 (19.7)
Deficiency anemia	4 (1.8)	488 (5.3)	353 (3.9)	855 (4.6)
Depression	15 (6.7)	757 (8.2)	1610 (17.7)	2399 (12.8)
Diabetes, complicated	99 (44.0)	2871 (31.2)	2099 (23.0)	5116 (27.3)
Diabetes, uncomplicated	58 (25.8)	1987 (21.6)	1673 (18.3)	3751 (20.0)
Drug abuse	4 (1.8)	144 (1.6)	116 (1.3)	264 (1.4)
Fluid and electrolyte disorders	127 (56.4)	4721 (51.4)	4640 (50.9)	9580 (51.1)
Hypertension, complicated	65 (28.9)	3182 (34.6)	2488 (27.3)	5768 (30.8)
Hypertension, uncomplicated	87 (38.7)	4299 (46.8)	4407 (48.3)	8875 (47.4)
Hypothyroidism	13 (5.8)	718 (7.8)	1583 (17.3)	2330 (12.4)
Kidney failure	61 (27.1)	2440 (26.5)	1725 (18.9)	4248 (22.7)
Liver disease	20 (8.9)	325 (3.5)	378 (4.1)	735 (3.9)
Lymphoma	0	61 (0.7)	78 (0.9)	139 (0.7)
Metastatic cancer	0	85 (0.9)	91 (1.0)	176 (0.9)
Obesity	61 (27.1)	3020 (32.9)	2143 (23.5)	5262 (28.1)
Other neurological disorders	41 (18.2)	1745 (19.0)	1683 (18.5)	3492 (18.6)
Paralysis	3 (1.3)	150 (1.6)	108 (1.2)	262 (1.4)
Peptic ulcer disease, excluding bleeding	0	25 (0.3)	22 (0.2)	47 (0.3)
Peripheral vascular disorders	2 (0.9)	447 (4.9)	495 (5.4)	949 (5.1)
Psychoses	2 (0.9)	277 (3.0)	166 (1.8)	445 (2.4)
Pulmonary circulation disorders	8 (3.6)	527 (5.7)	429 (4.7)	972 (5.2)
Rheumatoid arthritis/collagen vascular diseases	10 (4.4)	295 (3.2)	354 (3.9)	663 (3.5)
Solid tumor without metastasis	1 (0.4)	226 (2.5)	212 (2.3)	441 (2.3)
Valvular disease	5 (2.2)	297 (3.2)	408 (4.5)	718 (3.8)
Weight loss	9 (4.0)	686 (7.5)	636 (7.0)	1340 (7.1)

^a^ Includes Asian, Native Hawaiian or other Pacific Islander, and 2 or more races or ethnicities; therefore, totals are greater than row totals.

### Inpatient Mortality

Across all 5 comorbidity risk score strata, American Indian and Alaska Native adults experienced in-hospital mortality rates 2 to 3 times that of all other races (Table 3). Odds of in-hospital mortality among Black patients were 75% lower than among American Indian and Alaska Native patients (OR, 0.25 [95% CI, 0.18-0.34]); odds of in-hospital death among White patients were 77% lower (OR, 0.23 [95% CI, 0.16-0.31]). American Indian and Alaska Native patients with the lowest risk (score ≤0) had an adjusted probability of in-hospital death of 0.10 compared with 0.03 for Black patients (OR, 0.29 [95% CI, 0.10-0.82]) and 0.04 for White patients (OR, 0.37 [95% CI, 0.13-1.07]). The greatest adjusted probability of in-hospital death observed across all races was among American Indian and Alaska Native patients with an ECI score of 16 or greater at 0.69, compared with 0.28 among Black patients (OR, 0.16 [95% CI, 0.08-0.32]) and 0.25 among White patients (OR, 0.14 [95% CI, 0.07-0.27]). At this highest ECI level, nearly 7 of every 10 admitted American Indian and Alaska Native patients died prior to discharge compared with less than 3 of every 10 Black or White patients. For those with the lowest comorbidity risk (ECI score ≤0), 1 of every 10 American Indian and Alaska Native adults died while in the hospital with COVID-19 compared with 1 or fewer of every 25 Black or White adults. The adjusted probability of death increased at each increasing ECI score strata for all races with the exception of American Indian and Alaska Native patients. The adjusted probability of in-hospital mortality for American Indian and Alaska Native patients decreased from 0.43 for those with an ECI score of 6 to 10 to 0.38 for those with an ECI score of 11 to 15, increasing again to 0.69 for those at the highest ECI score strata of 16 or greater. Overall, American Indian and Alaska Native adults who died in the hospital with COVID-19 were younger (mean [SD], 58.0 [15.7] years) than all other races.

**Table 3.** Adjusted Associations of Inpatient Mortality Among Adults With COVID-19 and a Known Race in All Nonfederal Mississippi Hospitals by Weighted ECI Score, 2020

ECI score	Race	Adjusted probability^a^	AOR (95% CI)^b^
≤0	American Indian and Alaska Native	0.10	1 [Reference]
	Black	0.03	0.29 (0.10-0.82)^c^
	White	0.04	0.37 (0.13-1.07)
1-5	American Indian and Alaska Native	0.25	1 [Reference]
	Black	0.08	0.25 (0.12-0.49)^c^
	White	0.08	0.25 (0.12-0.50)^c^
6-10	American Indian and Alaska Native	0.43	1 [Reference]
	Black	0.16	0.24 (0.12-0.48)^c^
	White	0.15	0.21 (0.10-0.43)^c^
11-15	American Indian and Alaska Native	0.38	1 [Reference]
	Black	0.18	0.34 (0.17-0.67)^c^
	White	0.16	0.30 (0.15-0.59)^c^
≥16	American Indian and Alaska Native	0.69	1 [Reference]
	Black	0.28	0.16 (0.08-0.32)^c^
	White	0.25	0.14 (0.07-0.27)^c^

Abbreviations: AOR, adjusted odds ratio; ECI, Elixhauser Comorbidity Index.

^a^ Adjusted probability of inpatient mortality from multivariate logistic regression model including age, sex, race, and ECI score.

^b^ Adjusted OR of inpatient mortality from multivariate logistic regression model including age, sex, race, and ECI score.

^c^ *P* < .05.

## Discussion

This study provides previously unreported demographic and clinical characteristics, comorbidity risks, and mortality experiences of Mississippians hospitalized with COVID-19 across 103 hospitals in the state. Although increased comorbidity risk was associated with in-hospital death, mortality was not uniformly observed across races at any risk level. Racial disparities exist such that American Indian and Alaska Native adults with COVID-19 had longer hospital stays and were significantly more likely than Black or White adults to die while in the hospital, despite a lower mean comorbidity risk burden. Disparate COVID-19 mortality not being attributed to increased comorbidity burden is counter to most empirical findings and even mainstream media reports to date.

Although we do not discredit the role that comorbidities may play in COVID-19 outcomes, alternative contributing factors to disparate COVID-19 hospitalization and mortality outcomes among Indigenous populations must be considered. Discrimination, marginalization, inability to see preferred clinicians, and systemic underfunding of the Indian Health Service (IHS) have been widely cited as barriers to American Indian and Alaska Native individuals accessing care.^24,25^ If these barriers are overcome, Indigenous persons still receive worse care than White individuals for an estimated 40% of quality measures.^26^ The COVID-19 pandemic has further exposed these disparities.

Members of the Mississippi Band of Choctaw Indians were faced with many of the COVID-19 prevention challenges commonly cited as contributors to high infection rates among other Indigenous populations, including multigenerational households and limited remote work options. By the summer of 2020, the Mississippi State Department of Health found COVID-19 seropositivity rates at testing locations within Choctaw communities exceeded 18% among American Indian and Alaska Native individuals; just 7% of Black and 6% of White community members tested were found to be seropositive during this same period (Thomas E. Dobbs III, MD; email communication, December 2021).

Barriers to American Indian and Alaska Native individuals receiving COVID-19 treatment in Mississippi may have been compounded. Each of the 8 official communities of the Mississippi Band of Choctaw Indians are federally designated as both rural and medically underserved regions.^27,28^ The 20-bed Choctaw Health Center on the reservation serves as the sole IHS-affiliated hospital in the state.^29^ In the COVID-19 Systems of Care Plan enacted by the state, the facility was designated a level IV COVID-19 center.^30^ By definition, the principal role of level IV centers—the lowest acuity designation—is to provide isolation and stabilization of patients and to transfer individuals to the “appropriate higher level of care based on the clinical presentation of the patient”.^30^ The only level I COVID-19 Center in Mississippi is in Jackson,^30^ the state capital, approximately 75 miles from the Choctaw reservation and hospital. Only 1 of the 11 level II centers in the state is within closer proximity yet is still 58 miles away from the IHS facility.^30^

American Indian and Alaska Native individuals needing COVID-19 treatment beyond isolation or stabilization had to seek care at a non-IHS facility. Although care at IHS facilities is provided to tribe members at no cost, federal payment for care received outside the IHS system requires referral by an IHS clinician and validation that the care cannot be provided by an IHS facility.^31^ Even those expenses deemed eligible for reimbursement through the complex and often lengthy reimbursement process known as Purchased/Referred Care are often not paid due to limited funding.^31^ An estimated 40% of the health care needs of eligible American Indian and Alaska Native individuals are not covered by funds appropriated by the US federal government.^31^ In Mississippi—a state with one of the highest rates of adult poverty and lowest rates of health insurance coverage in the country—these additional barriers may create double disparity situations for American Indian and Alaska Native residents such that COVID-19 care is delayed or prevented altogether. Indigenous patients in the described study having the longest COVID-19 hospital admission times among residents of all races could reflect such delays.

Our finding that American Indian and Alaska Native adults, compared with Black or White adults, in Mississippi are both younger when hospitalized with COVID-19 and younger when dying in the hospital is consistent with study findings in other states. In Alaska, Arizona, and New Mexico, for instance, COVID-19 mortality rates among American Indian and Alaska Native persons were comparable to those of White individuals 20 to 30 years older.^24^ This reality has devastating repercussions on Indigenous populations who have already seen tremendous loss among tribal elders. Each death signifies not only the loss of life, but also the loss of tribal culture, tradition, and language.

### Limitations

This study has several limitations. First, its cross-sectional design does not allow for causal inferences. Also, only those individuals who sought medical care at a nonfederal hospital in Mississippi were included. Because these facilities exclusively practice western allopathic medicine, we cannot account for American Indian and Alaska Native individuals seeking traditional treatments. Given the markedly higher barriers that racial and ethnic minority groups experience in receiving care, they may be further underrepresented in our cohort. In addition, patient comorbidity risk scores were calculated by screening 2020 admissions data for *ICD-10* codes entered in the patient’s electronic health record. Patient histories were not available, potentially resulting in incomplete comorbidity profiles. Patient vaccination status was also not available. The first COVID-19 vaccinations were administered in Mississippi in mid-December 2020, however. Given that our data collection period ended December 31, 2020, we believe most, if not all, included patients were not yet vaccinated. This should result in more comparable comparisons of immune responses across patients without potentially conflating vaccine influences on mortality outcomes. Finally, American Indian and Alaska Native tribes and the social environments in which they exist are unique; generalizability of the described study findings to other Indigenous communities is limited for this reason.

## Conclusions

In this cross-sectional study of US adults hospitalized with COVID-19, American Indian and Alaska Native patients were (1) younger than patients of all other races when admitted, (2) younger than patients of all other races who died in the hospital, and (3) more likely to die in the hospital than adults of all other races, regardless of comorbidity burden. These findings have important public and population health implications. COVID-19 prevention and treatment strategies should be culturally specific, with policy changes seeking to address pervasive and systemic inequalities among Indigenous populations. Inclusion of American Indian and Alaska Native populations in public health reports and study findings, accurate racial and ethnic classification, and access to evidence-based treatments^32^ may serve as critical first steps. Additional investigations to understand alternative contributing factors to disparate COVID-19 experiences among American Indian and Alaska Native individuals must be prioritized as part of state and national health initiatives to improve health outcomes in the current pandemic and beyond.

## Notes

Supplement. **eTable.** Elixhauser Comorbidity Index (ECI) Risk Factors and Associated *ICD-10* Code(s)
