# Supplementary material for: In-Hospital Mortality Disparities Among American Indian and Alaska Native, Black, and White Patients With COVID-19
Source: JAMA Netw Open. 2022 Mar 30;5(3):e224822. doi: 10.1001/jamanetworkopen.2022.4822 (PMC8968465; doi:10.1001/jamanetworkopen.2022.4822)
Supplement: Supplement. — eTable. Elixhauser Comorbidity Index (ECI) Risk Factors and Associated ICD-10 Code(s) [file jamanetwopen-e224822-s001.pdf]

Supplementary Online Content

Musshafen LA, El-Sadek L, Lirette ST, Summers RL, Compretta C, Dobbs TE III. In-hospital mortality disparities among American Indian and Alaska Native, Black, and White patients with COVID-19. *JAMA Netw Open*. 2022;5(3):e224822.
doi:10.1001/jamanetworkopen.2022.4822

eTable. Elixhauser Comorbidity Index (ECI) Risk Factors and Associated *ICD-10* Code(s)

This supplementary material has been provided by the authors to give readers additional information about their work.

© 2022 Musshafen LA et al. *JAMA Network Open*.

eTable. Elixhauser Comorbidity Index (ECI) Risk Factors and Associated *ICD-10* Code(s)

**ECI Risk Factor**	**Associated *ICD-10* Code(s)**
AIDS/HIV	B20.x-B22.x, B24.x
Alcohol abuse	F10, E52, G62.1, I42.6, K29.2, K70.0, K70.3, K70.9, T51.x, Z50.2, Z71.4, Z72.1
Blood loss anemia	D50.0
Cardiac arrhythmias	I44.1-I44.3, I45.6, I45.9, I47.x-I49.x, R00.0, R00.1, R00.8, T82.1, Z45.0, Z95.0
Chronic pulmonary disease	I27.8, I27.9, J40.x-J47.x, J60.x-J67.x, J68.4, J70.1, J70.3
Coagulopathy	D65-D68.x, D69.1, D69.3-D69.6
Congestive heart failure	I09.9, I11.0, I13.0, I13.2, I25.5, I42.0, I42.5-I42.9, I43.x, I50.x, P29.0
Deficiency anemia	D50.8, D50.9, D51.x-D53.x
Depression	F20.4, F31.3-F31.5, F32.x, F33.x, F34.1, F41.2, F43.2
Diabetes, complicated	E10.2-E10.8, E11.2-E11.8, E12.2-E12.8, E13.2-E13.8, E14.2-E14.8
Diabetes, uncomplicated	E10.0, E10.1, E10.9, E11.0, E11.1, E11.9, E12.0, E12.1, E12.9, E13.0, E13.1, E13.9, E14.0, E14.1, E14.9
Drug abuse	F11.x-F16.x, F18.x, F19.x, Z71.5, Z72.2
Fluid and electrolyte disorders	E22.2, E86.x, E87.x
Hypertension, complicated	I11.x-I13.x, I15.x
Hypertension, uncomplicated	I10.x
Hypothyroidism	E00.x-E03.x, E89.0
Liver disease	B18.x, I85.x, I86.4, I98.2, K70.x, K71.1, K71.3-K71.5, K71.7, K72.x-K74.x, K76.0, K76.2 - K76.9, Z94.4
Lymphoma	C81.x-C85.x, C88.x, C90.0, C90.2, C96.x,
Metastatic cancer	C77.x-C80.x
Obesity	E66.x
Other neurological disorders	G10.x-G13.x, G20.x-G22.x, G25.4, G25.5, G31.2, G31.8, G31.9, G32.x, G35.x-G37.x, G40.x, G41.x, G93.1, G93.4, R47.0, R56.x
Paralysis	G04.1, G11.4, G80.1, G80.2, G81.x, G82.x, G83.0-G83.4, G83.9
Peptic ulcer disease, excluding bleeding	K25.7, K25.9, K26.7, K26.9, K27.7, K27.9, K28.7, K28.9
Peripheral vascular disorders	I70.x, I71.x, I73.1, I73.8, I73.9, I77.1, I79.0, I79.2, K55.1, K55.8, K55.9, Z95.8, Z95.9
Psychoses	F20.x, F22.x-F25.x, F28.x, F29.x, F30.2, F31.2, F31.5
Pulmonary circulation disorders	I26.x, I27.x, I28.0, I28.8, I28.9
Renal failure	I12.0, I13.1, N18.x, N19.x, N25.0, Z49.0-Z49.2, Z94.0, Z99.2
Rheumatoid arthritis/collagen vascular diseases	L94.0, L94.1, L94.3, M05.x, M06.x, M08.x, M12.0, M12.3, M30.x, M31.0-M31.3, M32.x-M35.x, M45.x, M46.1, M46.8, M46.9
Solid tumor without metastasis	C00.x-C26.x, C30.x-C34.x, C37.x-C41.x, C43.x, C45.x-C58.x, C60.x-C76.x, C97.x
Valvular disease	A52.0, I05.x - I08.x, I09.1, I09.8, I34.x-I39.x, Q23.0-Q23.3, Z95.2-Z95.4
Weight loss	E40.x-E46.x, R63.4, R64

© 2022 Musshafen LA et al. *JAMA Network Open*.
